# Estimated Methicillin-Resistant *Staphylococcus aureus* Decolonization in Intensive Care Units Associated With Single-Application Chlorhexidine Gluconate or Mupirocin

**DOI:** 10.1001/jamanetworkopen.2021.0652

**Published:** 2021-03-04

**Authors:** Eric T. Lofgren, Matthew Mietchen, Kristen V. Dicks, Rebekah Moehring, Deverick Anderson

**Affiliations:** 1 Paul G. Allen School for Global Animal Health, Washington State University, Pullman, Washington; 2 Duke Center for Antimicrobial Stewardship and Infection Prevention, Durham, North Carolina

## Abstract

**Importance:**

Chlorhexidine gluconate (CHG) and mupirocin are widely used to decolonize patients with methicillin-resistant *Staphylococcus aureus* (MRSA) and reduce risks associated with infection in hospitalized populations. Quantifying the association of an application of CHG alone or in combination with mupirocin with risk of MRSA infection is important for studies evaluating alternative decolonization strategies or schedules and for identifying whether there is room for improved decolonizing agents.

**Objective:**

To estimate the proportion of patients with MRSA decolonized per application of CHG and mupirocin from existing population-level studies.

**Design, Setting, and Participants:**

A stochastic mathematical model of an 18-bed intensive care unit (ICU) in an academic medical center operating over 1 year was used to estimate parameters for the proportion of simulated patients with MRSA decolonized per application of CHG and mupirocin. The model was conducted using approximate bayesian computation with data from an existing meta-analysis of studies conducted from February 2005 through January 2015. Data were analyzed from January 2018 through November 2019.

**Exposure:**

A universal decolonization protocol for colonized patients in the ICU using CHG or CHG and mupirocin in combination was simulated.

**Main Outcomes and Measures:**

The proportion of patients with MRSA decolonized per application of CHG and mupirocin was estimated.

**Results:**

The estimated proportion of patients with MRSA decolonized per application of CHG was 0.15 (95% credible interval, 0.01-0.42), and the estimated proportion per application of mupirocin in conjunction with CHG was 0.15 (95% credible interval, 0.01-0.54). A lag in colonization detection was associated with decreases in the CHG estimate (0.11; 95% credible interval, 0.01-0.30) and mupirocin estimate (0.10; 95% credible interval, 0.00-0.34), which were sensitive to the value of the modeled contact rate between nurses and patients. A 1% increase in the value of this parameter was associated with a 0.73% increase in the estimated combined outcomes associated with CHG and mupirocin (95% CI: 0.71, 0.75). Gaps longer than 24 hours in the administration of decolonizing agents were associated with a decrease of within-ICU MRSA transmission. Compared with a mean (SD) of 1.23 (0.27) acquisitions per 1000 patient-days in scenarios with no decolonizing bathing, a bathing protocol administering CHG and mupirocin every 120 hours was associated with a mean (SD) acquisition rate of 1.03 (0.24) acquisitions per 1000 patient days, a 16.3% decrease (95% CI, 14.7%-18.0%; *P* > .001).

**Conclusions and Relevance:**

These findings suggest that there may be room for significant improvement in anti-MRSA disinfectants, including the compounds themselves and their delivery mechanisms. Despite the decolonization estimates found in this study, these agents are associated with robust outcomes after delays in administration, which may help in alleviating concerns over patient comfort and toxic effects.

## Key Points

**Question:**

What is the estimated proportion of patients with methicillin-resistant *Staphylococcus aureus* (MRSA) decolonized per application of chlorhexidine and mupirocin?

**Findings:**

This mathematical modeling study estimated that chlorhexidine and mupirocin were independently associated with a 15% likelihood of decolonization in patients with MRSA.

**Meaning:**

This study found that, on a per-application basis, chlorhexidine and mupirocin were associated with decolonization; this suggests that there may be room for improvement and innovation in MRSA decolonization interventions.

## Teaser

This mathematical modeling study estimates the decolonization of methicillin-resistant *Staphylococcus aureus* (MRSA) associated with chlorhexidine gluconate and mupirocin in a simulated intensive care unit (ICU).

## Introduction

Despite recent progress in decreased incidence of methicillin-resistant *Staphylococcus aureus* (MRSA) in hospitals,^1^ it remains a targeted pathogen for infection prevention and public health efforts. One intervention with increasingly widespread use is decolonization of patients with MRSA using chlorhexidine gluconate (CHG) baths for the skin and mupirocin for the nares. While a 2016 randomized clinical trial^2^ found these interventions to be effective, the results from a 2016 study^3^ based primarily in community hospital intensive care units (ICUs) were more equivocal. There are several possible explanations for this discrepancy. The results from the randomized clinical trial may not generalize well to settings with lower MRSA incidence or with differing levels of mupirocin resistance. Similarly, lower-incidence settings may not be sufficiently powered to detect an association between implementing decolonization programs and decolonization outcomes. Finally, changes in implementation from the trial setting to everyday use may be associated with decreases in the overall outcome of the intervention. Thus, infection prevention programs considering these strategies in lower-incidence settings must justify the cost of decolonization products and implementation efforts in their hospitals. A better understanding of outcomes associated with the per-patient application level may help programs weigh the implementation costs of these interventions.

Evaluating these discrepancies requires a mechanistic understanding of the outcomes associated with decolonization; that is, what is the probability of decolonization if a patient is treated with a decolonization agent? This estimate is essential for a number of potential uses: cost-effectiveness studies, quantifying the outcomes associated with a decolonization protocol in conjunction with other interventions, or studying future changes in outcomes, owing to new technology, emerging resistance to decolonizing agents, or other factors. Obtaining such an estimate empirically, especially in a community setting, would be difficult, requiring intensive and repeated sampling of patients with already complicated clinical presentations. Rather than directly measuring the probability of successful decolonization, a mathematical modeling approach can estimate the proportion of patients with MRSA who are decolonized per application of CHG and mupirocin; the model can estimate the proportion that best supports results seen in clinical trials and can estimate the degree of certainty. The aim of this mathematical modeling study was to estimate this quantity for CHG bathing and CHG bathing in conjunction with mupirocin decolonization of the nares.

## Methods

Given that this mathematical modeling study used already published data, Washington State University’s institutional review board (IRB) determined that the study was not human participant research and did not require IRB review or informed consent under US federal regulation (45 CFR part 46.102). This study followed the Modeling Infectious Diseases (MIND) in Healthcare reporting guidelines.

### MRSA Transmission Model

**Figure 1.**
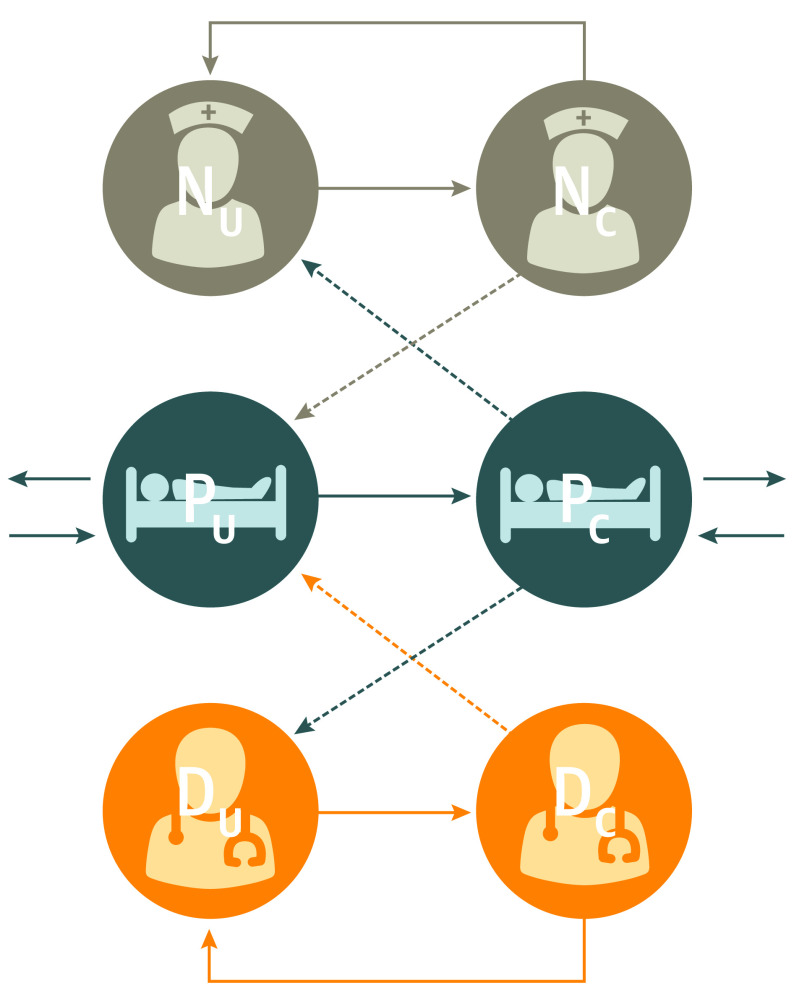
Compartmental Flowchart Solid arrows indicate possible transition states; dashed arrows, potential routes of methicillin-resistant *Staphylococcus aureus* contamination or colonization; NU, nurses who are uncontaminated; DU, doctors who are uncontaminated; NC, nurses who are contaminated; DC, doctors who are contaminated; PU, patients who are uncolonized; PC, patients who are colonized.

We adapted stochastic compartmental models published in 2019^4^ and 2017^5^ of transmission of MRSA through an ICU. Our model included compartments for patient colonization status and the presence or absence of contamination on the hands or clothing of health care workers (HCWs). Patients were modeled as presently uncolonized (P_U_) or colonized (P_C_), while HCWs were modeled as uncontaminated (S_U_) or contaminated (S_C_). The model assumed that transmission occurred when an HCW who was contaminated came into contact with a patient who was uncolonized, and contamination occurred when an HCW who was uncontaminated came into contact with a patient who was colonized (Figure 1). Given that there is considerable evidence that MRSA can be spread via surface contamination as well as via direct contact,^6^ we modeled contact between a patient and an HCW as a direct-care task^7,8^ involving interaction with a patient or the patient’s immediate surroundings. We made a simplifying assumption that there were no contamination pathways directly from HCW to HCW.

**Figure 2.**
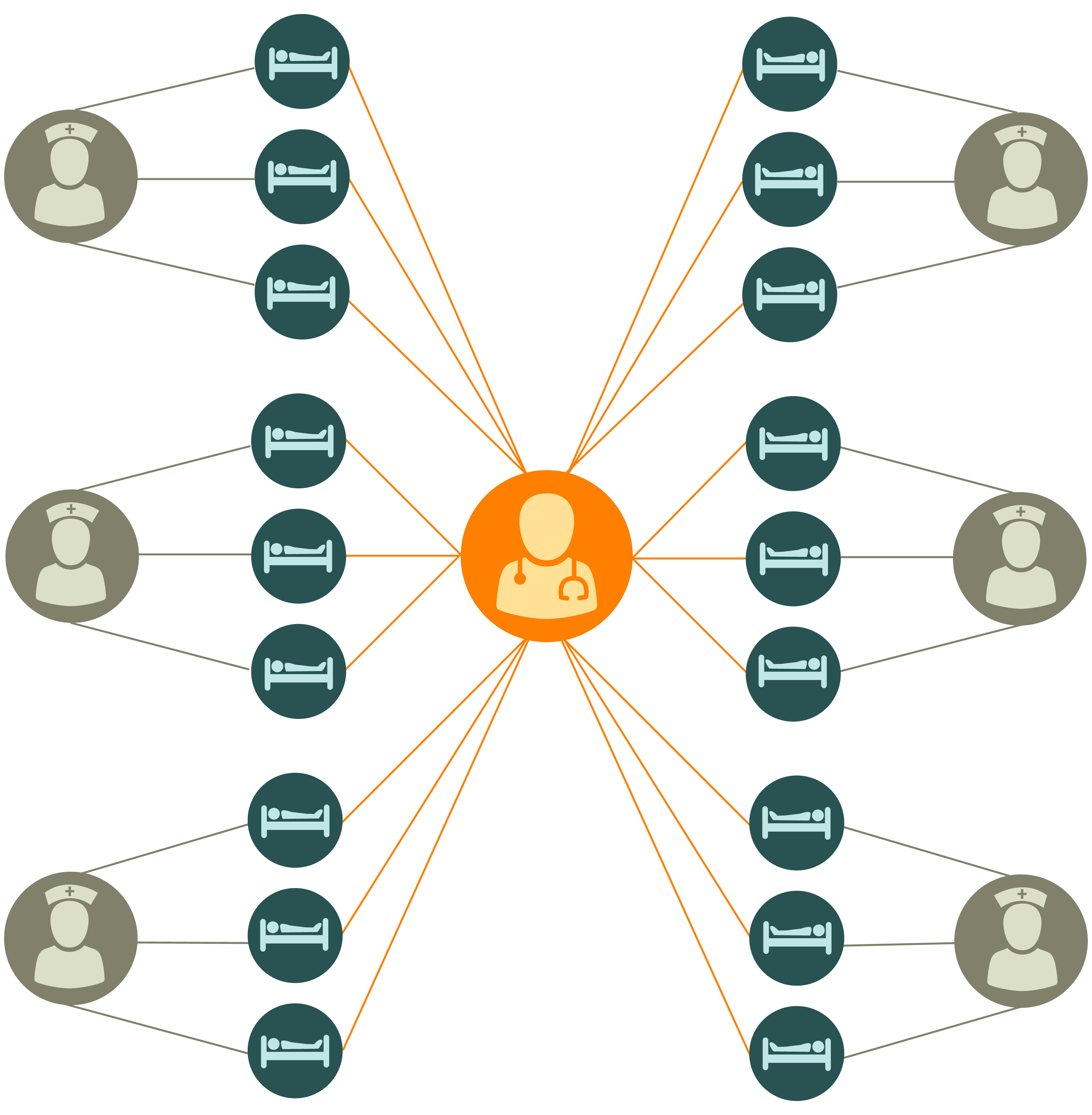
Schematic of a Structured Intensive Care Unit Population Model Patients (blue) are treated by a single assigned nurse (gray). A single intensivist (orange) randomly treats all patients.

We simulated a single year for an 18-bed, closed ICU assumed to be at maximum capacity with a 1:3 nurse-patient ratio and a single dedicated intensivist. Because an ICU is a highly structured population, this model relaxed the random mixing assumption used in many compartmental models, instead subdividing the patient population such that each patient was cared for by a single nurse who exclusively cared for 3 patients. While this is a simplification of the structure of an ICU, a 2019 study^4^ found that this assumption was associated with more conservative outcomes compared with random mixing. The intensivist was assumed to treat all patients (Figure 2).

This model made several simplifying assumptions intended to mimic the environment of a hospital with no major gaps in its infection control program. Patients were assumed to be homogeneous in their risk of MRSA acquisition, and the contact frequency between HCWs and patients, while nonrandom, was uniform (ie, there were no patients who were particularly difficult or contact intensive). It was assumed that patients did not interact with each other directly and were assigned to single-occupancy rooms. Hospitals were assumed to follow standard contact precaution guidelines set forward by the Centers for Disease Control and to detect MRSA colonization with perfect accuracy. While this is a strong assumption clinically, it is associated with decreased changes in outcomes given that the intervention is being applied universally. Finally, all HCWs were assumed to wash their hands after each direct-care task and to change their gloves and gowns at the same rate at which they entered and exited patient rooms.

### Parameterization and Model Calibration

The model used parameter values from models published in 2019^4^ and 2017.^5^ The values of each parameter in the model and the sources^7,8,9,10,11,12,13^ they were drawn from are detailed in the Table. The stochastic reaction equations used in the model and the code necessary to run the simulations are available at GitHub.^14^ Technical details of the model following the MIND Healthcare model description framework^15^ are given in eMethods in the Supplement. The transition terms are provided in the eTable in the Supplement. Where possible, parameters were drawn from studies from large academic medical centers similar to the ones conducting large randomized clinical trials on decolonization protocols.

**Table.** Model Parameters

Parameter	Parameter description	Parameter value	Sources
ρ	Contact rate between patients and HCWs	4.154 (No. of direct care tasks/h)	Westbrook et al,^7^ 2011; Lofgren et al,^10^ 2014
ρ_N_	Contact rate between patients and nurses	3.973 (No. of nurse direct care tasks/h)	Westbrook et al,^7^ 2011; Lofgren et, al^10^ 2014
ρ_D_	Contact rate between patients and physician	0.181 (No. of physician direct care tasks/h)	Westbrook et al,^7^ 2011; Lofgren et al,^10^ 2014
σ	Probability that a HCW’s hands are contaminated from a single contact with a patient who is colonized	0.054	Austin et al,^11^ 1999
ψ	Probability of successful colonization of a patient who is uncolonized owing to contact with an HCW who is contaminated in metapopulation structure	0.4481	Fitted to Harris et al,^9^ 2013
θ	Probability of discharge	4.39 d^−1^	Harris et al,^9^ 2013
ν_u_	Proportion of admitted patients who are uncolonized with MRSA	0.9221	Harris et al,^9^ 2013
ν_c_	Proportion of admitted patients who are colonized with MRSA	0.0779	Harris et al,^9^ 2013
ι	Effective hand decontaminations/h (direct care tasks × hand hygiene compliance × efficacy)	5.740 (10.682 direct care tasks/h ×56.55% compliance × ~ 95% efficacy)	Westbrook et al,^7^ 2011; Harris et al,^9^ 2013; Lofgren et, al^10^ 2014; Sickbert-Bennett et al,^12^ 2005
ι_N_	Effective nurse hand-decontaminations/h	6.404 (11.92 direct care tasks/h × 56.55% compliance × ~ 95% efficacy)	Westbrook et al,^7^ 2011; Harris et al,^9^ 2013; Lofgren et, al^10^ 2014; Sickbert-Bennett et al,^12^ 2005
ι_D_	Effective physician hand-decontaminations/h	1.748 (3.253 direct care tasks/h × 56.55% compliance × ~ 95% efficacy)	Westbrook et al,^7^ 2011; Harris et al,^9^ 2013; Lofgren et, al^10^ 2014; Sickbert-Bennett et al,^12^ 2005
τ	Effective gown or glove changes/h (2 × No. of visits × compliance)	2.445 (2.957 changes/h × 82.66% compliance)	Ballermann et al,^8^ 2011; Harris et al,^9^ 2013; Austin et al,^11^ 1999
τ_N_	Effective nurse gown or glove changes/h	2.728 (3.30 changes/h × 82.66% compliance)	Ballermann et al,^8^ 2011; Harris et al,^9^ 2013; Austin et al,^11^ 1999
τ_D_	Effective physician gown or glove changes/h	0.744 (0.90 changes/h × 82.66% compliance)	Ballermann et al,^8^ 2011; Harris et al,^9^ 2013; Austin et al,^11^ 1999
μ	Natural decolonization rate	20.0 d^−1^	Khader et al,^13^ 2017
δ	Per-application outcome associated with CHG	Estimated	NA
ζ	Per-application outcome associated with CHG plus mupirocin	Estimated	NA
η	Decolonization application frequency	24.0 h^−1^	NA

Abbreviations: CHG, chlorhexidine gluconate; HCW, health care worker; MRSA, methicillin-resistant *Staphylococcus aureus*; NA, not applicable.

### Decolonization Intervention Outcome Estimation

To estimate the proportion of patients with MRSA decolonized per application of CHG and mupirocin, we used a 3-step fitting procedure: baseline, intervention 1 (ie, CHG baths), and intervention 2 (ie, CHG baths plus nasal mupirocin). First, a baseline model of a preintervention ICU was fit using a single free parameter, ψ, for the probability that a patient who was uncolonized became colonized after contact with an HCW who was contaminated. This parameter was tuned such that the model had a mean (SD) incidence of 5.89 (1.35) MRSA acquisitions per 1000 patient-days.^5,9^ Second, we introduced a new parameter, δ, to represent CHG-based (ie, intervention 1) decolonization, when a patient moved from a colonized status to an uncolonized status. This parameter was assumed to be associated with a 0.748 incidence rate ratio compared with the baseline model, in line with a meta-analysis of CHG-only studies by Kim et al.^2^ Third, a second parameter, ζ, moved patients from colonized to uncolonized status and was fit to represent the addition of nasal decolonization with mupirocin accompanying a CHG bathing protocol (ie, intervention 2); it was assumed to be associated with a combined incident rate ratio of 0.578. This formulation assumed that the outcomes associated with CHG and mupirocin were additive and that there were no synergistic associations between them.

Approximate bayesian computation (ABC) was used to fit these parameters. Details on this method for model fitting may be found elsewhere.^16,17^ Briefly, it is a computational technique that draws a candidate value from a prior distribution, simulates the model using that value, and accepts the candidate value if the simulated result is within an error band around a given summary statistic. In this case, we fit the model to incidence rates associated with the baseline and the 2 simulated interventions. The distribution of these accepted values is an approximation of a bayesian posterior. In this study, all parameters were fit using 1 000 000 parameter draws from a uniform prior distribution bounded by 0 and 1.0 followed by a single iteration of the simulation. Candidate parameters were accepted with an error term, ε = 0.05, indicating that the simulated incidence rates had to be from 5% less than to 5% greater than the target incidence rate on the log scale. The primary strength of this technique is that it allows for the leveraging of clinical information in the form of summary statistics from the literature, in the form of both the prior and the acceptance criteria, even when the original study data are unavailable.

### Statistical Analysis

We conducted 3 sensitivity analyses. The first varied the frequency with which decolonization was applied, comparing a baseline of no decolonization with applications of CHG and mupirocin every 24, 48, 72, 96, and 120 hours to determine if a significant component of the modeled proportion is associated with the typical schedule of a daily CHG bath.

The second was a global sensitivity analysis, simultaneously allowing each parameter to vary uniformly by 50% more than or less than its original value. For each parameter draw, 200 model runs were performed and the joint association of CHG and mupirocin (as a single parameter) was reestimated. This process was repeated 5000 times, and the distribution of the parameter values examined to ensure adequate coverage. Linear regression was used to estimate the relative association of a single percentage change in each parameter value with the estimated proportion of patients with MRSA decolonized per application of CHG and mupirocin.

Finally, we conducted a structural sensitivity analysis examining the change in outcomes associated with assuming, as our model did, that there was no latent period in MRSA colonization, wherein a patient was colonized at subdetectable levels. We added a latent period to our model, wherein patients transitioned from P_s_ to a new compartment (ie, P_E_, representing latent colonization) before finally transitioning to P_c_, the colonized state. The rate of transition from P_E_ to P_c_ was varied randomly from 1 to 4 days.^18^ It was assumed that patients in P_E_ did not shed sufficient amounts of MRSA to contaminate HCWs. This created a small pool of patients who were decolonized owing to treatment but were not credited as such, thus underestimating the association between treatment and decolonization. The proportion of patients with MRSA decolonized per application of CHG and mupirocin was then reestimated using the same procedures as in the main model.

*P* values were 2-sided, and *α* = .05 was considered significant unless otherwise specified. Data were analyzed from January 2018 through November 2019 using R statistical software version 4.02 (R Project for Statistical Computing).

## Results

### Per-Application Outcomes Associated With CHG Bathing and CHG-Mupirocin Combinations

**Figure 3.**
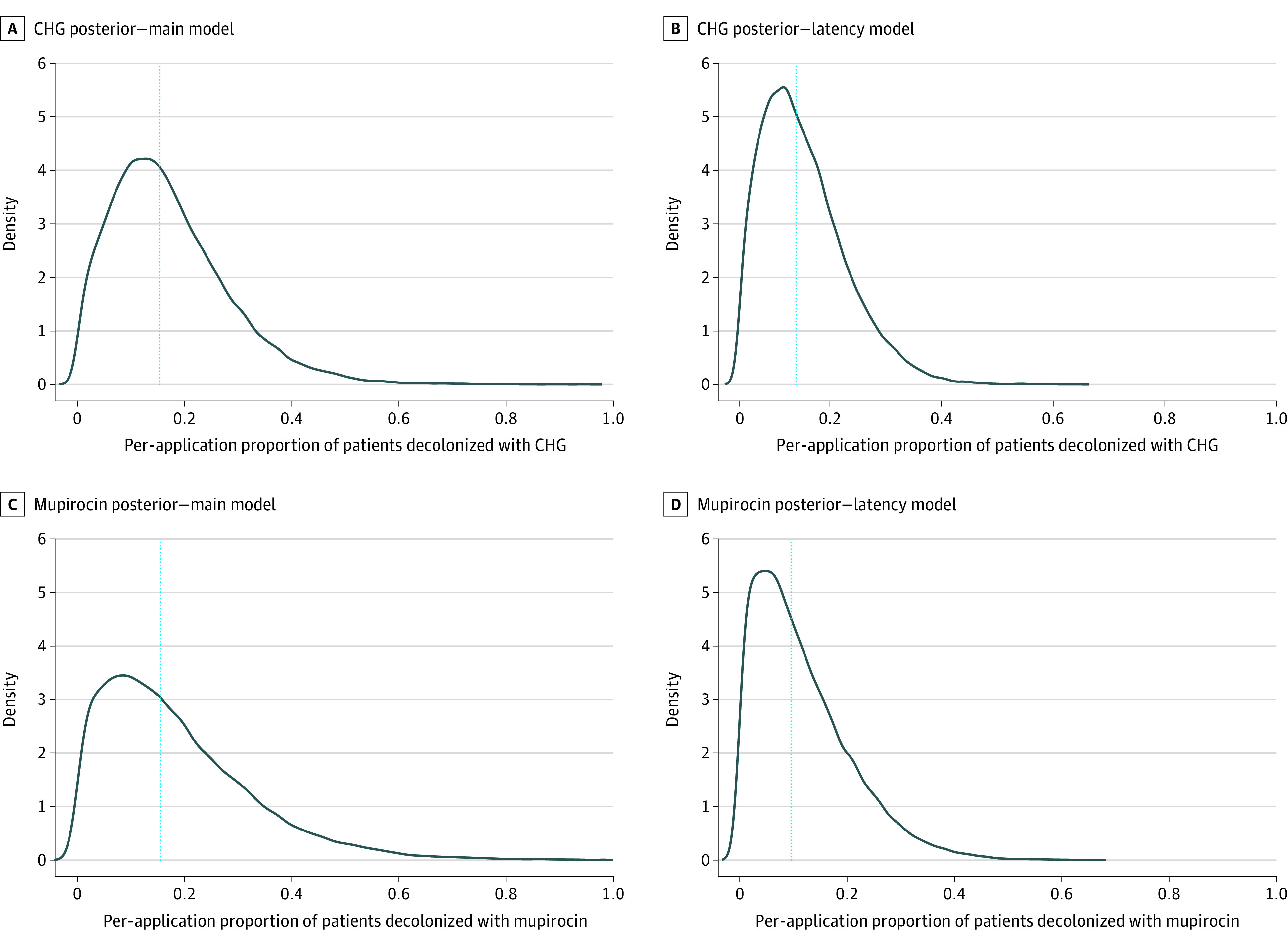
Estimates of Per-Application Proportion of Patients Decolonized Curves indicate density (estimated using a normal kernel) of values for chlorhexidine gluconate (CHG; ie, δ) or mupirocin (ie, ζ); vertical dotted lines, median of this density; left-hand panels, estimates assuming acquisition is instantly detected; right-hand panels, estimates assuming a 1-day to 4-day latent period in which a patient may be colonized or decolonized but the individual’s acquisition is not yet detected.

To achieve colonization rates similar to those seen in Kim et al,^2^ the estimated proportion of patients with MRSA decolonized per application of CHG was 0.15 (95% credible interval, 0.01-0.42), meaning less than one-sixth of applications of CHG were expected to be associated with decolonization of the patient. Mupirocin’s estimated proportion was 0.15 (95% credible interval, 0.01-0.54) when used in conjunction with an existing CHG-based intervention. The posterior densities of the efficacy estimates are shown in Figure 3. The addition of a 1-day to 4-day latent period between the transmission event and recognized MRSA colonization was associated with a decrease in the estimates. In this case, the proportion of patients with MRSA decolonized was estimated to be 0.11 (95% credible interval, 0.01-0.30) per application of CHG and 0.10 (95% credible interval, 0-0.34) per application of mupirocin with CHG (Figure 3).

### Model Sensitivity to Variation in Timing and Parameter Uncertainty

**Figure 4.**
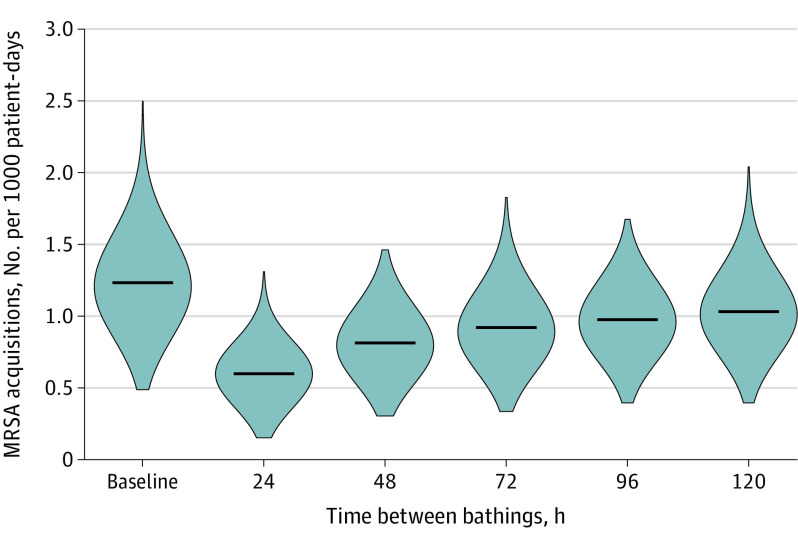
Sensitivity of Decolonization Protocols to Changes in Timing Violins indicate smoothed kernel-density estimates of 1000 runs of the model with a given timing for the administration of decolonizing baths; horizontal bars, mean estimates for each scenario; MRSA, methicillin-resistant *Staphylococcus aureus*.

Despite this per-application estimate of outcomes, the timing sensitivity analysis found that decreases in MRSA acquisitions were associated with bathing even at less frequent bathing intervals. Compared with a mean (SD) of 1.23 (0.27) acquisitions per 1000 patient-days in scenarios with no bathing, a bathing protocol administering CHG and mupirocin every 120 hours (ie, 5 days) was associated with a mean (SD) acquisition rate of 1.03 (0.24) acquisitions per 1000 patient days, a 16.3% decrease (95% CI, 14.7%-18.0%; *P* > .001) (Figure 4).

The model’s results were most sensitive to the value of ρ_Ν_ , the contact rate between nurses and patients. A 1% increase in the value of this parameter was associated with a 0.73% increase in the estimated combined efficacy of CHG and mupirocin (95% CI, 0.71%-0.75%). Other sensitive parameters included ψ, the probability of colonization given contact between an HCW who is contaminated and a patient (0.43%; 95% credibility interval, 0.41%-0.44%), and ν, the proportion of admitted patients colonized with MRSA (0.37%; 95% CI, 0.35%-0.39%). The sensitivity estimates for all varied parameters are shown in the eFigure in the Supplement.

## Discussion

Using a mathematical model to translate from population-level effect estimates to a per-application estimate of the proportion of patients decolonized, this study found that on a per-application basis the estimated proportion of patients with MRSA decolonized was 0.15 for CHG and mupirocin in conjunction with CHG. Under ideal circumstances, the combination of the 2 compounds was estimated to be associated with decolonization 30% of the time. Under more realistic circumstances, in which there was some delay and uncertainty between the acquisition and detection of MRSA owing to biological processes associated with colonization or laboratory testing, this estimate decreased to 20%.

These results should not be taken as a condemnation of the utility of CHG or mupirocin as options for reducing the transmission of MRSA within hospitals. Rather, they suggest that even relatively imperfect interventions may still be associated with decreased transmissions owing to decreased colonization pressure and as part of a suite of imperfect interventions implemented by infection control, the so-called Swiss cheese approach. Furthermore, these results suggest that there may be room for further gains by improving the methods by which we decolonize patients. Importantly, this model cannot determine whether the proportion of patients with MRSA decolonized per application of CHG and mupirocin is associated with the compounds themselves or the way in which they are applied. This means that even in the absence of novel compounds, improvements to the methods of applying CHG and mupirocin may be associated with significant benefits.^19,20^

The results of the timing-focused sensitivity analysis suggest that considerable deviations from an intensive 24-hour decolonization schedule may still be associated with decreases in the unit-level MRSA acquisition rate. Most health care–associated pathogens have relatively low transmissibility,^20,21,22^ so any decrease in the colonization pressure within an ICU, even a modest one, may interrupt delicate transmission chains. The results of this study suggest that deviations from a daily CHG bathing schedule owing to concerns over toxic effects in neonatal populations, patient-reported skin irritation, or other practical demands may still be useful interventions. These results may also be useful for future studies, allowing facilities to estimate the expected outcomes associated with using these interventions in their specific settings. This may allow for the critical evaluation of existing studies and provide clear estimates that can be used to estimate the outcomes associated with resistance to CHG, mupirocin, or both by the addition of compartments representing patients who are colonized with resistant organisms. These estimates may also be useful for future studies examining the complex interplay between interventions that target the pathway between contact with a contaminated patient or that individual’s environment and subsequent colonization of another patient. Those interventions may include hand hygiene, environmental cleaning, and other measures. The outcomes associated with each of these interventions may vary and may be associated with the level of the others.

When comparing these results with the experiences of real-world health care settings, a number of things must be considered, most notably the sensitivity of the model to other parameters. Settings with significantly lower hand hygiene rates (compared with the modeled rate of 56.55%) or personal protective equipment use rates would have higher estimates of CHG and mupirocin decolonization, given that they have more potential contamination events to clear. Settings with a higher proportion of patients admitted with preexisting MRSA colonization, owing to those locations’ catchment populations or other factors, would also have higher estimates than those presented in this study.

### Limitations

This mathematical modeling study has several limitations. It assumes that the ICU in the model, which is meant to represent the type of academic medical center in which large-scale intervention trials are most often conducted, is a reasonable representation of the environment in which the studies were conducted. The parameter sensitivity analysis found that the model is most sensitive to errors in the contact rate between nurses and patients. Furthermore, this study made similar assumptions as the meta-analysis by Kim et al^2^ that was used to calibrate the model and estimate the per-application outcomes associated with CHG and mupirocin. That is, this study assumed that the studies in question, a mix of randomized clinical trials and interrupted time-series studies, were capable of estimating the population-level outcomes associated with decolonization without bias. Additionally, this model assumed that there were no cumulative benefits associated with repeated bathing; each application was treated as an independent event.

## Conclusions

This mathematical modeling study found that the proportion of patients with MRSA decolonized was 0.15 per application of CHG and per application of mupirocin alongside CHG. The study represents an innovative use of mathematical modeling to translate population-level studies of a hospital epidemiology intervention using summary statistics to estimate an individual-level association. In particular, the proportion of patients with MRSA decolonized using these compounds would be difficult, if not impossible, to directly measure in a working health care setting. This study suggests that there may be opportunities for improved decolonization interventions associated with further decreases in MRSA rates in the ICU and that there may be room for deviation from intensive daily protocols in response to patient or clinician needs without jeopardizing their outcomes.

## Notes

Supplement. **eMethods.** Modeling Infectious Diseases in Healthcare Model Description Framework **eTable.** Transitions and Equations for Metapopulation Model of Acquisition and Decolonization **eFigure.** Global Sensitivity of Mathematical Model of Acquisition and Decolonization **eReferences**
