# Supplementary material for: Estimated Methicillin-Resistant Staphylococcus aureus Decolonization in Intensive Care Units Associated With Single-Application Chlorhexidine Gluconate or Mupirocin
Source: JAMA Netw Open. 2021 Mar 4;4(3):e210652. doi: 10.1001/jamanetworkopen.2021.0652 (PMC7933999; doi:10.1001/jamanetworkopen.2021.0652)
Supplement: Supplement. — eMethods. Modeling Infectious Diseases in Healthcare Model Description Framework eTable. Transitions and Equations for Metapopulation Model of Acquisition and Decolonization eFigure. Global Sensitivity of Mathematical Model of Acquisition and Decolonization eReferences [file jamanetwopen-e210652-s001.pdf]

Supplemental Online Content

Lofgren ET, Mietchen M, Dicks KV, Moehring R, Anderson D; Centers for Disease Control Modeling Infectious Diseases in Healthcare Program. Estimated methicillin-resistant *Staphylococcus aureus* decolonization in intensive care units associated with single-application chlorhexidine gluconate or mupirocin. *JAMA Netw Open*. 2021;4(3):e210652. doi:10.1001/jamanetworkopen.2021.0652

eMethods. Modeling Infectious Diseases in Healthcare Model Description Framework
eTable. Transitions and Equations for Metapopulation Model of Acquisition and Decolonization
eFigure. Global Sensitivity of Mathematical Model of Acquisition and Decolonization
eReferences

This supplemental material has been provided by the authors to give readers additional information about their work.

© 2021 Lofgren ET et al. *JAMA Network Open.*

eMethods. Modeling Infectious Diseases in Healthcare Model Description Framework

Purpose and Scope:
Purpose: The purpose of this model is the estimation of the per-application effectiveness of two MRSA decolonizing compounds (Chlorhexidine Gluconate and Mupirocin) based on site-level effect estimates found in the empirical literature.

Scope: A single 18-bed intensive care unit in a U.S. academic medical center. This ICU is represented as a closed ICU, with no interaction with the rest of the hospital.

Entities, state variables, and scales:
Entities: Patients, Nurses and Doctors. Interaction is defined entirely based on potentially contaminating/transmitting interactions. As a result, patients interact with nurses and doctors but not each other, while nurses and doctors both interact with patients but, again, not with each other. Nurses are specifically restricted to only interact with their assigned patient group.

State Variables: Patients are classified as Uncolonized, Colonized, or Latent (a delay state between the transmission event and detectable colonization). Patients are further segregated into five distinct groups, representing patients assigned to a particular nurse.

Nurses and Doctors are both represented as being either Uncontaminated or Contaminated and are identified individually within the model.

Scale: An 18-bed intensive care unit simulated for one year.

Initialization:
In the initial state of the model (at time = 0), there are six uncontaminated nurses, one uncontaminated doctor, and six groups of three patients, all of whom are uncolonized. Further discussion of the effects of varying this initial state may be found in[1]. No burn-in period was used in the model as visual inspection suggested this initial state was relatively close to the stochastic equilibrium of the model.

Process Overview and Scheduling:
A full description of the processes of the model may be found in the eTable, and for brevity are not presented here. The model is simulated using Gillespie's Direct Method[2], which selects the time the next event of any type occurs, and then randomly determines what type of event occurs based on their respective rates. As such, there is no overlying scheduling structure.

Input Data:
The model uses no external input data to represent processes in the model.

Agent interactions and organism transmission:

© 2021 Lofgren ET et al. *JAMA Network Open.*

Interactions: Interactions are event driven, and concentrated on the interactions between healthcare workers (HCWs) and patients. At a given rate per hour ρ_N for nurses and ρ_D for doctors, HCWs engage in a “direct care task”[3] which involves touching the patient or their immediate surrounding environment.

This interaction prompts many other possible events in the model, including pathogen transmission (described below), HCW hand/body contamination, hand washing, and the donning/doffing of PPE by HCWs.

Patients *do not* interact with other patients directly – all patient-to-patient conflict is modeled as indirect interactions via shared and contaminated HCWs.

Pathogen Transmission:
Pathogen transmission is entirely indirect. A patient who is colonized (or has contaminated their environment) can contaminate a HCW they have come into contact with. If this HCW does not clear this contamination either by washing their hands or by removing contaminated PPE, there is a per-direct care task probability (ψ) that an uncolonized patient will be successfully colonized, representing a within-healthcare facility transmission event.

Stochasticity:
Due to the model’s implementation using Gillespie’s Direct Method[2], the times events occur, and which event triggers at a given time are fully stochastic in the model. All other elements of the model, such as population size and parameter values, are deterministic.

Submodels: This model has no submodels.

Model verification, calibration and validation:

Verification: The model’s code was based on a previously published model. All code used in the model was subject to code review, and several extreme value tests (setting particular parameter values to very high or very low values that should subsequently result in implausible results) were conducted.

Calibration and Validation: The baseline model with no intervention was calibrated to the control arm of a large MRSA-related RCT[4] to produce an average incidence of 5.89 MRSA acquisitions per 1000 patient-days, as in a previously published study[5]. The parameters for both CHG and Mupirocin were estimated from sub-analyses of a meta-analysis published by Kim *et al.*[6] such that the parameter values for those interventions would, on average, cause a relative reduction in acquisitions matching the value of the meta-analysis. This was done using Approximate Bayesian Computation, drawing 1,000,000 candidate values for each parameter from a uniform prior bounded by 0 and 1 with an error term $\varepsilon = 0.05$, meaning that a candidate value would be accepted if the simulated incidence rate using that parameter fell within 5% of the targeted incidence rate on the log scale.

© 2021 Lofgren ET et al. *JAMA Network Open.*

eTable. Transitions and Equations for Metapopulation Model of Acquisition and Decolonization

Transition	Equation
N_{C_i} to N_{U_i}	$\iota_N N_{Ci}$; $i = 1 \dots 6$
N_{C_i} to N_{U_i}	$\tau_N N_{Ci} \frac{P_{Ci}}{(P_{Ci} + P_{Ui})} \gamma$; $i = 1 \dots 6$
N_{C_i} to N_{U_i}	$\tau_N N_{Ci} \frac{P_{Cj}}{(P_{Cj} + P_{Uj})} [(1-\gamma)/5]$; $i = 1 \dots 6, j = 1 \dots 6, j \neq i$
N_{U_i} to N_{C_i}	$\rho_N \sigma N_{Ui} \frac{P_{Ci}}{(P_{Ci} + P_{Ui})} \gamma$; $i = 1 \dots 6$
N_{U_i} to N_{C_i}	$\rho_N \sigma N_{Ui} \frac{P_{Cj}}{(P_{Cj} + P_{Uj})} [(1-\gamma)/5]$; $i = 1 \dots 6, j = 1 \dots 6, j \neq i$
D_C to D_U	$\iota_D D_C$
D_C to D_U	$\tau_D D_C \frac{\sum_{i=1}^{6} P_{Ci}}{\sum_{i=1}^{6} (P_{Ci} + P_{Ui})}$
D_U to D_C	$\rho_D \sigma D_U \frac{\sum_{i=1}^{6} P_{Ci}}{\sum_{i=1}^{6} (P_{Ci} + P_{Ui})}$
P_{U_i} to P_{C_i}	$\rho_N \psi P_{Ui} \frac{N_{Ci}}{(N_{Ci} + N_{Ui})} \gamma$; $i = 1 \dots 6$
P_{U_i} to P_{C_i}	$\rho_N \psi P_{Ui} \frac{N_{Cj}}{(N_{Cj} + N_{Uj})} [(1-\gamma)/5]$; $i = 1 \dots 6, j = 1 \dots 6, j \neq i$
P_{U_i} to P_{C_i}	$\rho_D \psi P_{Ui} \frac{D_C}{(D_C + D_U)}$; $i = 1 \dots 6$
P_{U_i} Discharge to P_{U_i} Admission	$\theta \nu_U P_{Ui}$; $i = 1 \dots 6$
P_{U_i} Discharge to P_{C_i} Admission	$\theta \nu_C P_{Ui}$; $i = 1 \dots 6$
P_{C_i} Discharge to P_{U_i} Admission	$\theta \nu_U P_{Ci}$; $i = 1 \dots 6$
P_{C_i} Discharge to P_{C_i} Admission	$\theta \nu_C P_{Ci}$; $i = 1 \dots 6$
P_{C_i} to P_{U_i}	μP_{Ci} ; $i = 1 \dots 6$
P_{C_i} to P_{U_i}	$\delta \eta P_{Ci}$; $i = 1 \dots 6$
P_{C_i} to P_{U_i}	$\zeta \eta P_{Ci}$; $i = 1 \dots 6$

© 2021 Lofgren ET et al. *JAMA Network Open.*

eFigure. Global Sensitivity of Mathematical Model of Acquisition and Decolonization

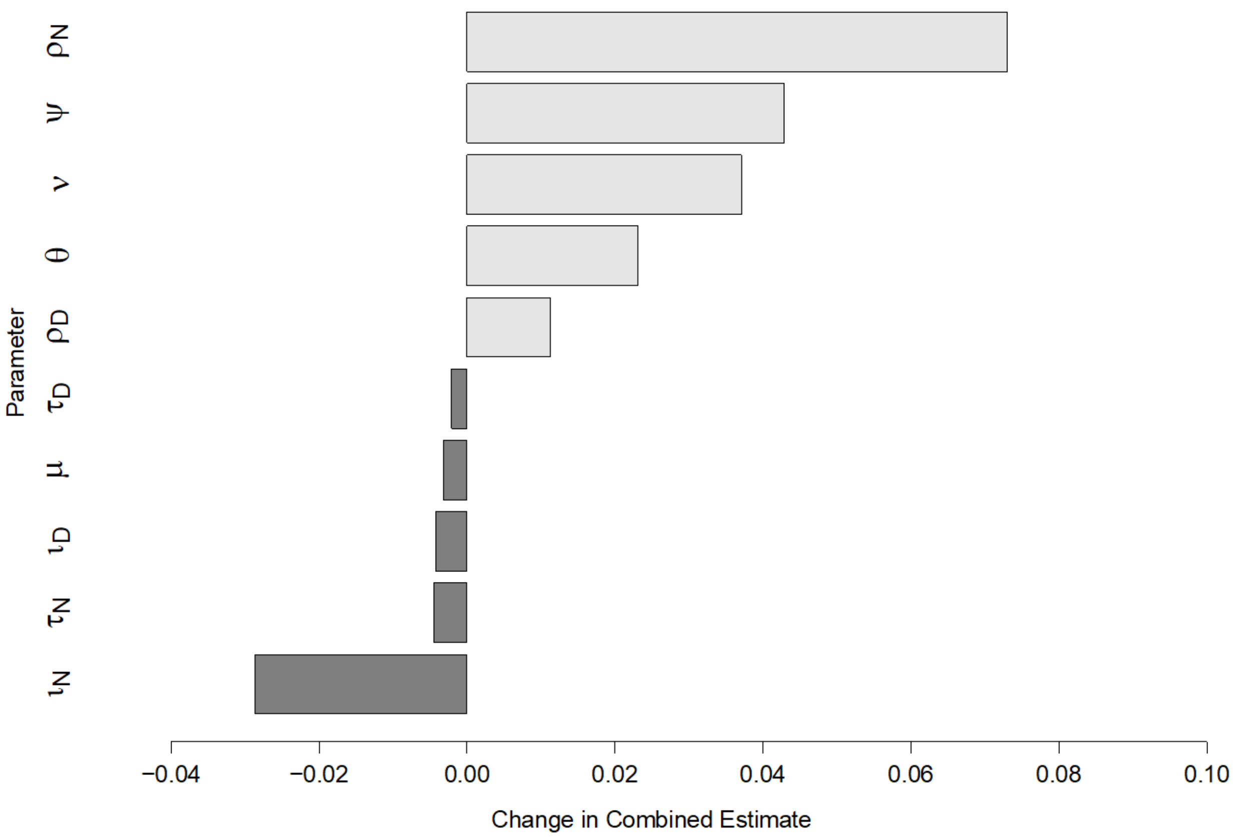

Horizontal bars represent the change in the estimated effectiveness of CHG/mupirocin decolonization per one-percent change in the value of a specific parameter, with light bars indicating increased estimated effectiveness and dark bars indicating decreased estimated effectiveness.

© 2021 Lofgren ET et al. *JAMA Network Open.*

eReferences

1. Short CT, Mietchen MS, Lofgren ET. Transient Dynamics of Infection Transmission in a Simulated Intensive Care Unit. *ArXiv190911878 Q-Bio*. Published online September 26, 2019. Accessed June 11, 2020. http://arxiv.org/abs/1909.11878

2. Gillespie DT. Exact stochastic simulation of coupled chemical reactions. *J Phys Chem*. 1977;81(25):2340–2361. doi:10.1021/j100540a008

3. Ballermann MA, Shaw NT, Mayes DC, Gibney RTN, Westbrook JI. Validation of the Work Observation Method By Activity Timing (WOMBAT) method of conducting time-motion observations in critical care settings: an observational study. *BMC Med Inform Decis Mak*. 2011;11:32. doi:10.1186/1472-6947-11-32

4. Harris AD, Pineles L, Belton B, et al. Universal glove and gown use and acquisition of antibiotic-resistant bacteria in the ICU: a randomized trial. *JAMA*. 2013;310(15):1571–1580. doi:10.1001/jama.2013.277815

5. Lofgren ET. Estimating the impact post randomization changes in staff behavior in infection prevention trials: a mathematical modeling approach. *BMC Infect Dis*. 2017;17(1):539. doi:10.1186/s12879-017-2632-1

6. Kim HY, Lee WK, Na S, Roh YH, Shin CS, Kim J. *The Effects of Chlorhexidine Gluconate Bathing on Health Care-Associated Infection in Intensive Care Units: A Meta-Analysis*.; 2016. doi:10.1016/j.jcrc.2015.11.011

© 2021 Lofgren ET et al. *JAMA Network Open.*
